# PAX6, a novel target of miR-335, inhibits cell proliferation and invasion in glioma cells

**DOI:** 10.3892/mmr.2021.12299

**Published:** 2021-07-16

**Authors:** Quan Cheng, Hui Cao, Zigui Chen, Zhiming Ma, Xin Wan, Renjun Peng, Bing Jiang

Mol Med Rep 10: 399-404, 2014; DOI: 10.3892/mmr.2014.2150

Following the publication of this paper, it was drawn to the Editors’ attention by a concerned reader that the western blotting data shown in Fig. 1B were strikingly similar to data appearing in different form in other articles by different authors. Owing to the fact that the contentious data in the above article had already been published elsewhere, or were already under consideration for publication, prior to its submission to *Molecular Medicine Reports*, the Editor has decided that this paper should be retracted from the Journal. After having been in contact with the authors, they agreed with the decision to retract the paper. The Editor apologizes to the readership for any inconvenience caused.

